# Revisiting nonlinearity of heart rate variability in healthy aging

**DOI:** 10.1038/s41598-023-40385-1

**Published:** 2023-08-14

**Authors:** Martín Calderón-Juárez, Gertrudis Hortensia González-Gómez, Juan C. Echeverría, Claudia Lerma

**Affiliations:** 1 International Collaboration on Repair Discoveries, Faculty of Medicine, University of British Columbia, Vancouver, BC V5Z 1M9 Canada; 2 Department of Electromechanical Instrumentation, Instituto Nacional de Cardiología Ignacio Chávez, Juan Badiano 1, Col. Sección 16, Tlalpan, 14080 Mexico City, Mexico; 3 Department of Physics, Faculty of Science, Universidad Nacional Autónoma de México, Ciudad de México, Mexico; 4 Department of Electrical Engineering, Universidad Autónoma Metropolitana Unidad Iztapalapa, Mexico City, Mexico; 5 Division of Physical Medicine and Rehabilitation, Department of Medicine, University of British Columbia, Vancouver, BC V5Z 2G9 Canada

**Keywords:** Computational biophysics, Data processing

## Abstract

Aging is commonly regarded as a physiological process in which the dynamic complexity of physiological time series and organ systems is gradually lost. This notion is derived from the identification of a decline of nonlinear measures with the advance of aging. However, additional research on cardiovascular control studied through heart rate variability (HRV), i.e., the instantaneous changes in heart rate, shows that despite the constriction of its statistical distribution, the nonlinear organization remains present in advanced age. Here, we used surrogate data testing to investigate the presence of nonlinear information in HRV time series from a publicly available database of 1121 healthy human subjects from 18 to 92 years old. We also studied the influence of basic clinical features, such as sex, body mass index (BMI), and mean heart rate (HR), on such nonlinear information. We found that the percentage of nonlinear time series after 30 years of age diminishes significantly (p < 0.01). Furthermore, larger BMI and HR are associated with the presence of more linear information in HRV, while the female sex is associated with the manifestation of nonlinear information. This work provides a common background for the contextualized interpretation of nonlinear testing and shows that the nonlinear content of HRV time series diminishes through aging.

## Introduction

It is widely considered that aging is related to a gradual loss of complexity in physiological function^1–3^. This notion comes from empirical observations in which the values of some complexity measures diminish with increasing age^2,4–6^, along with the continuous reduction in the variance and predominance of low-frequency components in heart rate variability (HRV)^4,7^. Contrary to the hypothesis that aging shows a decline in physiological function and a reduction of physiological complexity dynamics^8^, it has been proposed that, despite showing reduced variability, certain nonlinear structures in physiological behavior remain unchanged^8,9^. These two alternative hypotheses represent different notions relating to aspects of the physiological control mechanisms expected to change in the aging process in contrast to the impairments accompanying certain pathological conditions^10^.

Heart rate variability (HRV), one of the main physiological time series subjected to nonlinear analysis^11^, refers to the fluctuations in consecutive heartbeats, i.e., the instantaneous changes in heart rate or heart-period intervals^12^. A large amount of robust clinical research has been published in which the nonlinear analysis of HRV has proven to be a promising mathematical clinical tool for the identification of higher-risk cardiovascular patients and a powerful instrument for the study of the multisystemic and intricate physiology of heart rate regulation^11^. Nonlinear methods are frequently used to obtain relevant information on HRV beyond the traditional description of its statistical and power spectral properties. However, the existence of such nonlinear properties is not always formally shown. Thus, the risk of indiscriminately searching for nonlinear properties that are not rigorously confirmed in the HRV time series is a common pitfall in the investigation of the dynamics in these time series.

Surrogate data testing is a reliable technique for proving, from the statistical point of view, that a nonlinear feature does exist in the time series under study. A specific nonlinear measure, known as the discriminating statistic, is compared with the distribution of the same property determined in a collection of bootstrap-generated signals (surrogates), which are very similar to the original data but lack that tested nonlinear feature^13^. This methodology has been applied in several scenarios^14–19^. Still, the heterogeneity among sample populations complicates its interpretation^14–19^, since the influence of even the most basic characteristics on nonlinear dynamics, such as age, sex, and body mass, are not taken into consideration. Hence, the physiological and clinical interpretation of nonlinear characteristics of HRV is unattainable by the lack of a common background.

In this work, we tested the presence of nonlinear information in human HRV. We examined its manifestation during aging in female and male subjects, as well as the effect of body mass index (BMI). We employed the publicly available database “Autonomic Aging: A dataset to quantify changes of cardiovascular autonomic function during healthy aging”^20–22^ by analyzing the HRV obtained from ECG recordings of 1121 healthy individuals with an age range from 18 to 92 years^20^. We propose the use of the nonlinear analysis tool recurrence plots and its measures of complexity determinism (DET) and laminarity (LAM) as discriminative statistics^23^ to test HRV through a robust wavelet-based surrogate algorithm^24^. The algorithm was previously validated in short-term HRV time series^14^. DET and LAM are, respectively, measures of predictability and laminar (intermittent) states in a given system^25^; from the scope of short-term neural modulation of HRV by the autonomic nervous system, the increase in the value of these metrics is tightly related with parasympathetic withdrawal^26^. Here, we will use the gradual wavelet reconstruction algorithm to generate surrogates that preserve with remarkable fidelity the power spectrum, data distribution, and nonstationarity of time series^24^. This ensures that from a statistical point of view, we accurately identify nonlinear behavior in HRV.

## Methods

### Study design

In this retrospective study, we assessed the ECG recordings from the database “Autonomic Aging: a dataset to quantify changes of cardiovascular autonomic function during healthy aging”^20–22^, which contains data from 1,121 healthy volunteers recruited at Jena University Hospital. The database contains at least one ECG channel (lead II), age range, sex, and body mass index (BMI), and it is described elsewhere^20^. Briefly, we analyzed one ECG channel recorded at 1000 Hz from each participant. The ECG recording was taken in a quiet and fully shaded room with the temperature set at 22 °C. According to the database information, after subjects lied down comfortably on an examination tilt table, the electrodes were placed and subjects were instructed to avoid movement, yawning, or coughing. After a few minutes in such position, the instructor started the recording. The recording length ranged from 8 to 36 min. Our protocol was approved by the Research and Ethics Committee of our institution (protocol number 22-1309).

**Figure 1:**
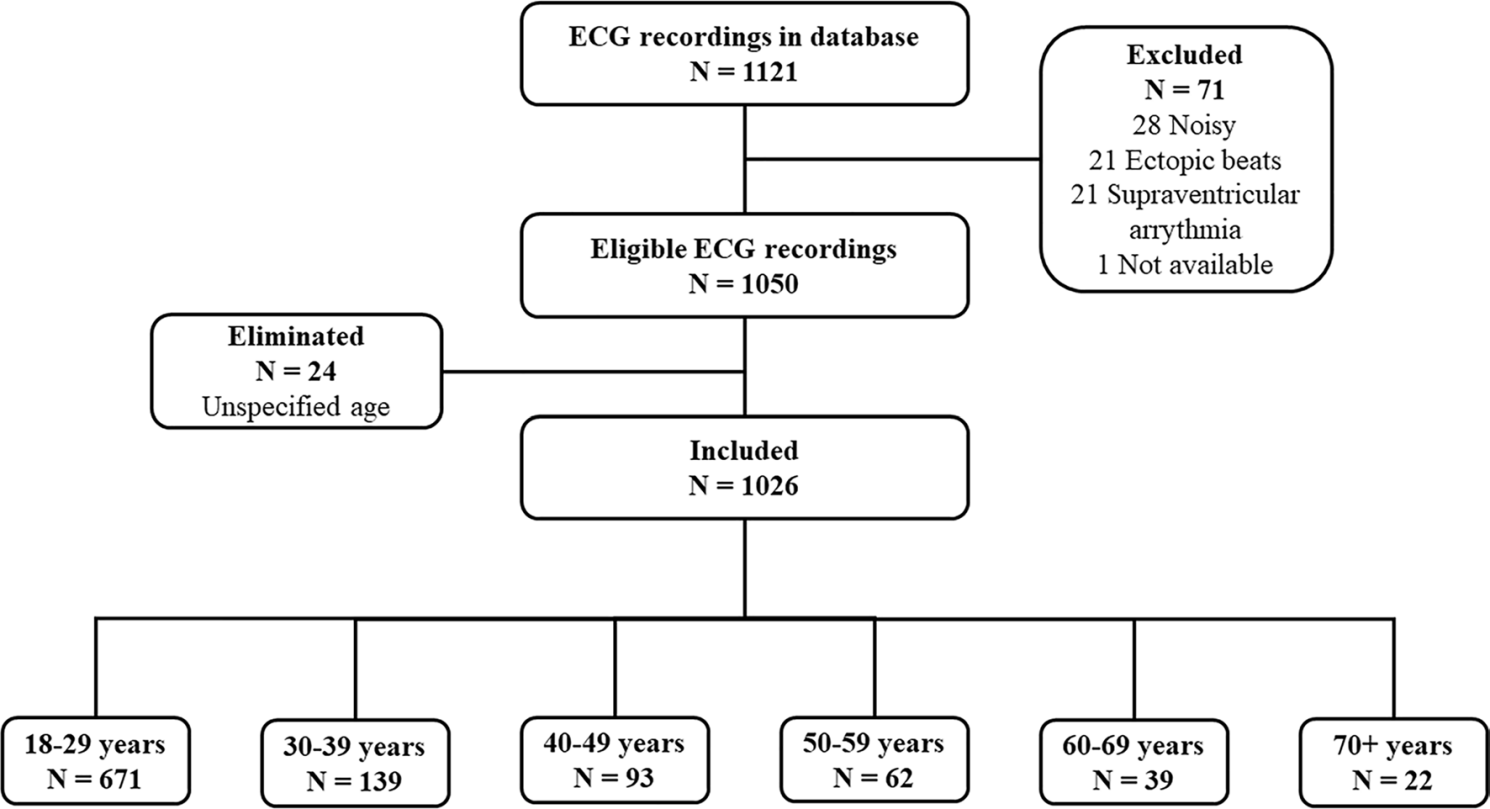
Selection of individuals included in the study.

Age groups are defined as follows: Group 1 (18–29 years), Group 2 (30–39 years), Group 3 (40–49 years), Group 4 (50–59 years), Group 5 (60–69 years) and Group 6 (70+ years), as they were presented elsewhere^20^. A segment of the last 5 min of each ECG recording was taken. If this segment was noisy or if any arrhythmia was observed, the 5-min segment nearest to the end was selected. Finally, if a 5-min segment free of arrhythmias, noise, or artifacts could not be found, the ECG recording was discarded. Seventy-one ECG recordings were not included owing to either noise in the signal, observations of ectopic heartbeats, supraventricular arrhythmias, or the unavailability of the ECG recording. Twenty-four subjects were also excluded owing to unspecified age in the database. Finally, 1026 subjects were considered for the analysis of HRV. Figure 1 summarizes how these cases were selected, whilst Table 1 describes how groups were conformed. The proportion of male and female participants was different across the age groups (p < 0.001).

**Table 1:** Healthy participants included in the study, by age and sex.

Age group (years)	Females, N = 632 (61.6%)	BMI (kg/m^2^)	Males, N = 394 (38.4%)	BMI (kg/m^2^)
18–29	454 (67.7%)	22 ± 3	217 (32.3%)	23 ± 3
30–39	65 (46.8%)	23 ± 4	74 (53.2%)	25 ± 5
40–49	44 (47.3%)	27 ± 6	49 (52.7%)	26 ± 4
50–59	28 (45.2%)	29 ± 9	34 (54.8%)	27 ± 4
60–69	24 (61.5%)	26 ± 5	15 (38.5%)	27 ± 3
70+	17 (77.3%)	26 ± 3	5 (22.7%)	26 ± 2

### ECG processing

According to the database information^20–22^, the ECG recordings were obtained from either MP150 (ECG100C, BIOPAC systems inc., Golata, CA, USA) or Task Force Monitor system (CN Systems Medizintechnik GmbH, Graz AUT). The ECG recordings were visually inspected by two of our trained observers and the correct identification of R waves was visually supervised in the Kubios HRV premium software^27^. After correction of misidentifications, artifacts, and identification of arrhythmias, an automatic beat correction algorithm^3^ was applied to correct any remaining ectopic heartbeats that were replaced by interpolated RR values. Most of the RR time series did not require any ectopy correction, heartbeat interpolation was applied only in 15 recordings (1.5%), and the percentage of heartbeats corrected by the automatic beat correction algorithm was within 0.55% and 1.24%.

### HRV linear indices

To provide a common reference framework, we provide a description of traditional HRV indices, time and frequency-based measurements^12^. We report the mean heart rate (HR), the standard deviation of NN intervals (SDNN), and the square root of the mean of the squares of differences between adjacent NN intervals (RMSSD).

Power spectral density was calculated by the discrete Fourier transform (DFT). The HRV series were resampled at 3 Hz to obtain evenly spaced HRV time series. To reduce the spectral leakage owing to the discontinuities at the boundaries of the finite HRV time series, a 300 data-points Hamming window function was applied within the resampled HRV time series with 50% overlapping. Finally, we used the DFT to calculate the mean power at the low-frequency band (LF, [0.04–0.15 Hz]), and high-frequency band (HF, [0.15–0.4 Hz]).

A larger statistical variability in HRV (SDNN and RMSSD), as well as a lower HR, reflect short-term predominance of parasympathetic activity; the HF band reflects parasympathetic and breathing pace regulation on HRV, and the LF band shows a mix of sympathetic and parasympathetic activity. Therefore, the LF/HF ratio (reported here) is usually used to provide a quick and global reference to the predominance of sympathetic over parasympathetic balance^12^.

### Recurrence plot quantification analysis (RQA)

RQA is a tool for nonlinear analysis based on the study of the textures observed in recurrence plots and is suitable for short, nonstationary, and noisy time series, such as HRV^25^. The phase space of a multidimensional system can be reconstructed from a unidimensional time series (such as HRV), and a recurrence plot is the visualization of the approximate dynamical recurrences that occur in such phase space. The recurrence plot is formally defined by:

1 $${R}_{i,j}=\Theta \left({\varepsilon }_{i}-\| {\overrightarrow{x}}_{i}-{\overrightarrow{x}}_{j}\| \right), i,j=1,\dots ,N,$$

where ${\varepsilon }_{i}$ is the distance that defines the vicinity of the point ${\overrightarrow{x}}_{i}$, and $\| \cdot \|$ is the norm that defines the shape or criteria to define the vicinity. Given that HRV time series are often nonstationary, the fixed amount of neighbors (FAN) was used to capture the time series dynamics, where ${\varepsilon }_{i}$ is variable for each point, and the recurrence density was fixed at 0.07. FAN norm is intended for the analysis of nonstationary data and the recurrence neighborhood varies along the data to maintain the same recurrence density for all points. $\Theta (x)$ is the Heaviside function, if the distance between points ${\overrightarrow{x}}_{i}$ and ${\overrightarrow{x}}_{j}$ is shorter than ${\varepsilon }_{i}$, the point ${\overrightarrow{x}}_{j}$ lies within the recurrence vicinity and the number 1 is assigned (${R}_{i,j}=1$), otherwise a 0 is assigned. We chose the embedding delay value τ by taking the first local minimum of the average mutual information function. The embedding dimension $m$ was chosen with the false nearest-neighbors function, when it reached its first value at 0. This approach has been described previously for short-term HRV^14^.

The complexity measures determinism (DET) and laminarity (LAM) were calculated from the recurrence plot of each HRV time series. DET is the ratio of recurrence points that form diagonal structures. This measure reflects that a segment of the trajectory is rather close during n time steps to another segment of the trajectory at a different time^25^. LAM refers to the ratio between recurrence points forming the vertical structures and the entire set of recurrence points and represents the occurrence of laminar states in the system^23^.

### Nonlinearity testing

The Pinned Wavelet Iterative Amplitude Adjusted Fourier Transform (PWIAAFT) algorithm described by Keylock, C.^28^ conveys the basis of nonstationarity preservation in surrogate data. In this algorithm, the time series at issue is decomposed using maximal overlap discrete wavelet transform (MODWT). Then, the wavelet coefficients of greater energy are pinned (fixed), and on the remaining, the iterative amplitude adjusted Fourier transform (IAAFT) is applied to dismantle nonlinear dynamics. However, this procedure was later refined to improve the convergence of time series and received the name of gradual wavelet reconstruction (GWR)^24^. In the latter technique for generating surrogate data, a cubic Hermitian polynomial is used to interpolate between fixed values. The IAAFT and GWR algorithms described in^24^ are also shown in the Supplementary Information.

In this work, we used a threshold $\rho =0.01$ for GWR surrogate generation, as used previously for 5-min HRV time series^14^. This is a robust algorithm to synthesize surrogate time series that preserve the statistical properties of the original data, as well as the power spectral density distribution. Furthermore, its main advantage against Fourier ransform-based algorithms is the preservation of nonstationarity in the original data. Ninety-nine surrogates for every original HRV time series were generated to achieve the surrogate tests with the significance α level of 0.01. According to the percentile in which the discriminant statistic value is located, a p-value was given. A statistically significant result for the surrogates was considered when $p<0.05$ (i.e., the hypothesis about presence of nonlinear information in the original time series was accepted).

### Statistical analysis

Categorical variables are described as absolute frequency (and percentages) and were compared between groups using Pearson’s chi-squared test. Continuous variables are described as mean ± standard deviation and were compared between two groups by Student’s t-test and among several groups by two-way analysis of variance (ANOVA). In the latest, we applied two-levels pairwise comparisons (sex and age group) for our dependent variables (HR, SDNN, RMSSD, LF/HR ratio, DET and LAM), with post hoc adjustment of p-values using the Bonferroni method. We compared sex and all age groups to all others. To assess the influence of independent variables age group, sex, BMI, and HR on surrogate data testing (i.e., linear and nonlinear classification of HRV time series) we performed a logistic regression to estimate the odds ratio (OR, 95% confidence interval). Statistical tests were done using the Statistical Package for the Social Sciences (SPSS 26, IBM SPSS Inc., USA) Statistical significance was considered at p < 0.05.

## Results

**Figure 2:**
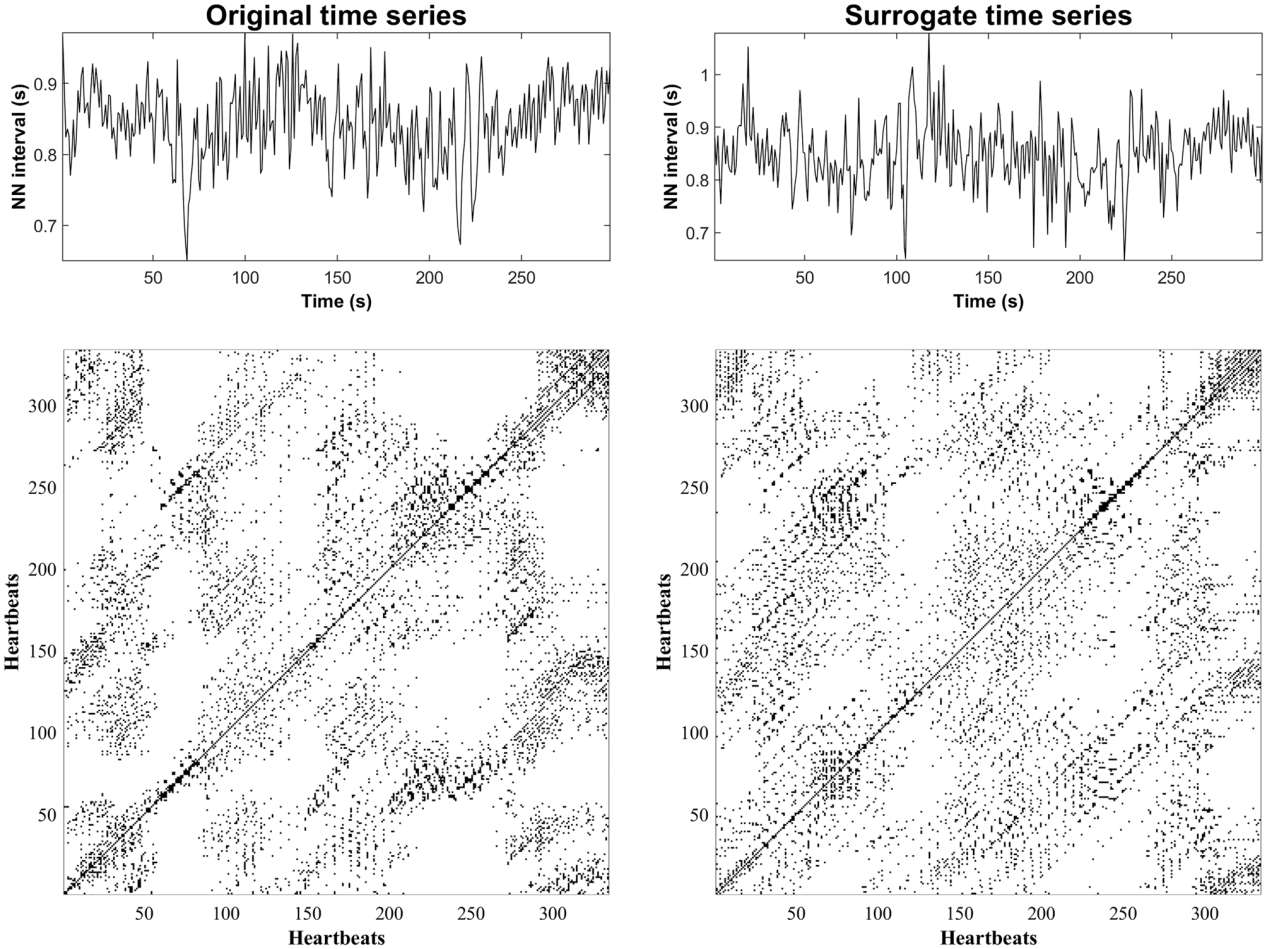
Illustrative HRV time series and a surrogate time series generated by the PWIAAFT algorithm (upper row). Also, their corresponding recurrence plots are shown (bottom row), where a similar texture is observed.

**Figure 3:**
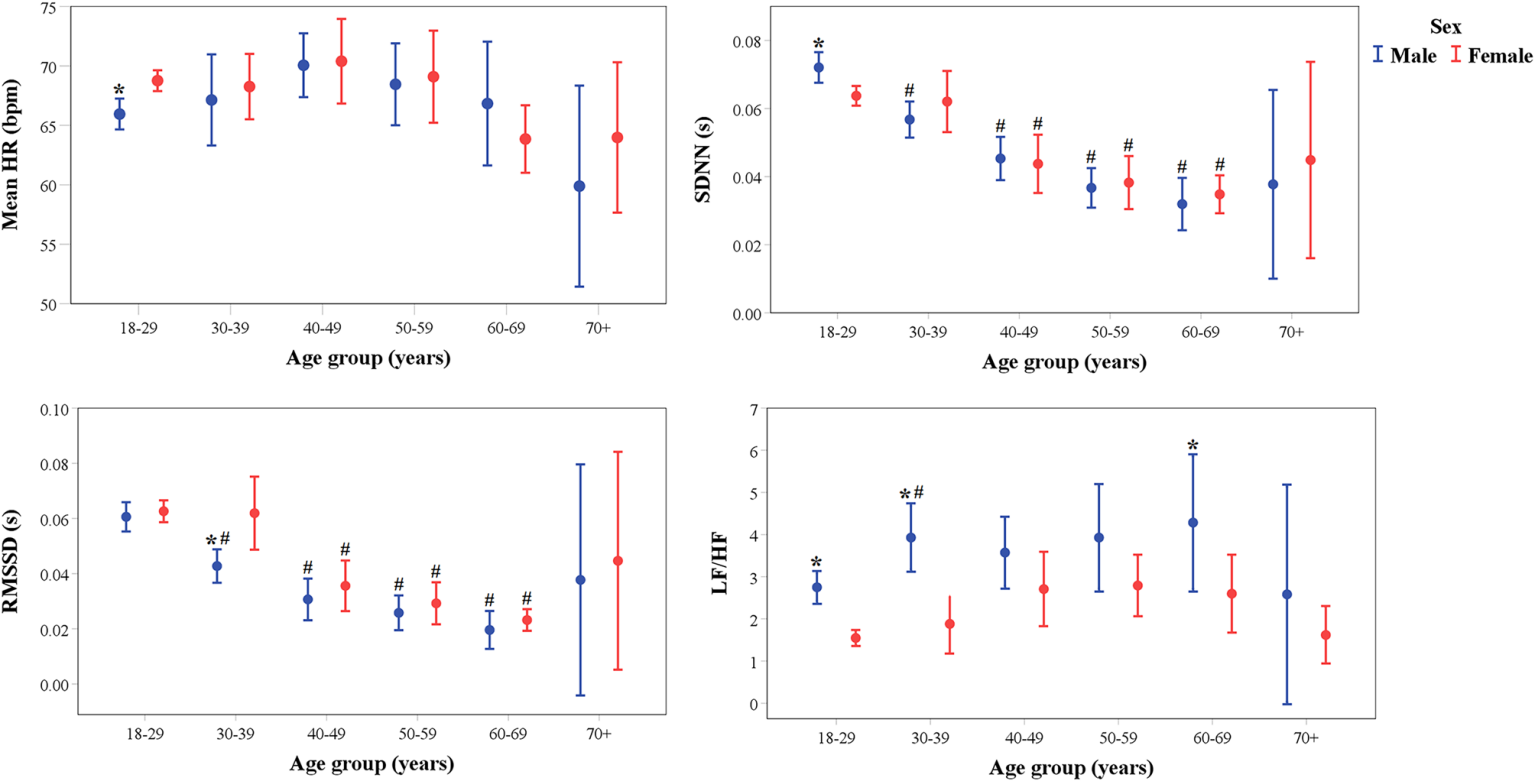
Heart rate variability linear indices grouped by age and sex. *p < 0.05 male vs. female (same age group). ^#^p < 0.05 vs. 18–29 years group (same sex).

Figure 2 illustrates the imitation of an original HRV time series by surrogate time series, as well as their corresponding recurrence plots. Figure 3 shows the results of HRV traditional indices through age groups. There are no significant differences in the mean HR of groups older than 30 years compared with the 18–29 years group. The groups older than 30 years of age show smaller values of SDNN and RMSSD compared with the 18–29 years group (same sex). Few differences are observed between males and females in all linear HRV indices (same age group).

**Figure 4:**
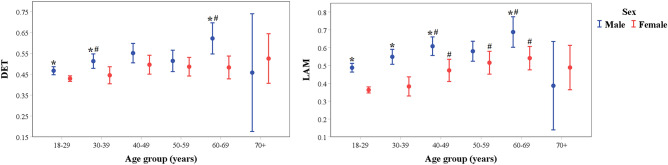
Recurrence quantitative analysis by age and sex. *p < 0.05 male vs. female (same age group). ^#^p < 0.05 vs. 18–29 years of age group (same sex).

Regarding RQA indices, few DET value differences were found along age groups and between males and females (Fig. 4). Larger values of LAM (Fig. 4) are observed in the groups of 40- 49, 50–59, and 60–69 years of age compared with the 18–29 years of age group in females and males. Also, larger values of LAM are observed in males compared with females from 18–69 years (except for the 50–59 years of age group).

Table 2 shows the p-values of ANOVA main effects and interactions. Age has a significant effect on all variables analyzed here. And sex has only a significant effect on LF/HF, DET and LAM. However, the interaction between age and sex is not significant for any HRV variable.

**Table 2:** Main ANOVA effects and interactions (p-values).

HRV variable	Main effects		Interaction (age × sex)
	Age	Sex	
HR	0.021	0.419	0.513
SDNN	< 0.001	0.750	0.172
RMSSD	< 0.001	0.159	0.387
LF/HF	< 0.001	< 0.001	0.387
DET	< 0.001	0.014	0.188
LAM	< 0.001	< 0.001	0.097

**Figure 5:**
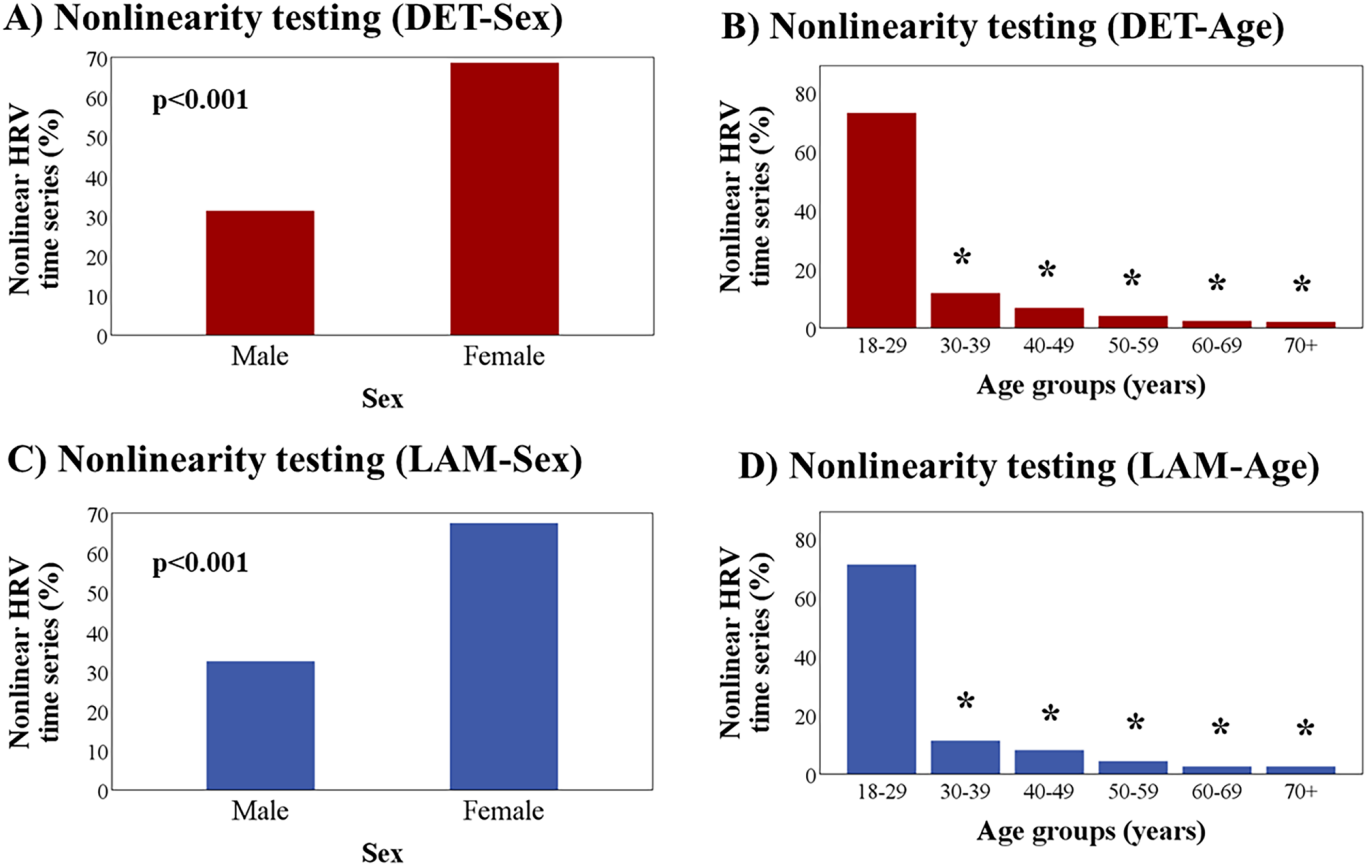
Percentage of nonlinear time series by sex (**A,C**) and age groups (**B,D**). *p < 0.001 vs. 18–29 years-old group.

A larger proportion of nonlinear time series was observed in the female group compared to the male group after surrogate data testing with DET and LAM as discriminating statistics (Fig. 5). Also, a smaller proportion of nonlinear time series was found in groups 30–39, 40–49, 50–59, 60–69, and 70+ years of age compared with the 18–29 years of age group with both discriminating statistics (Fig. 5).

**Figure 6:**
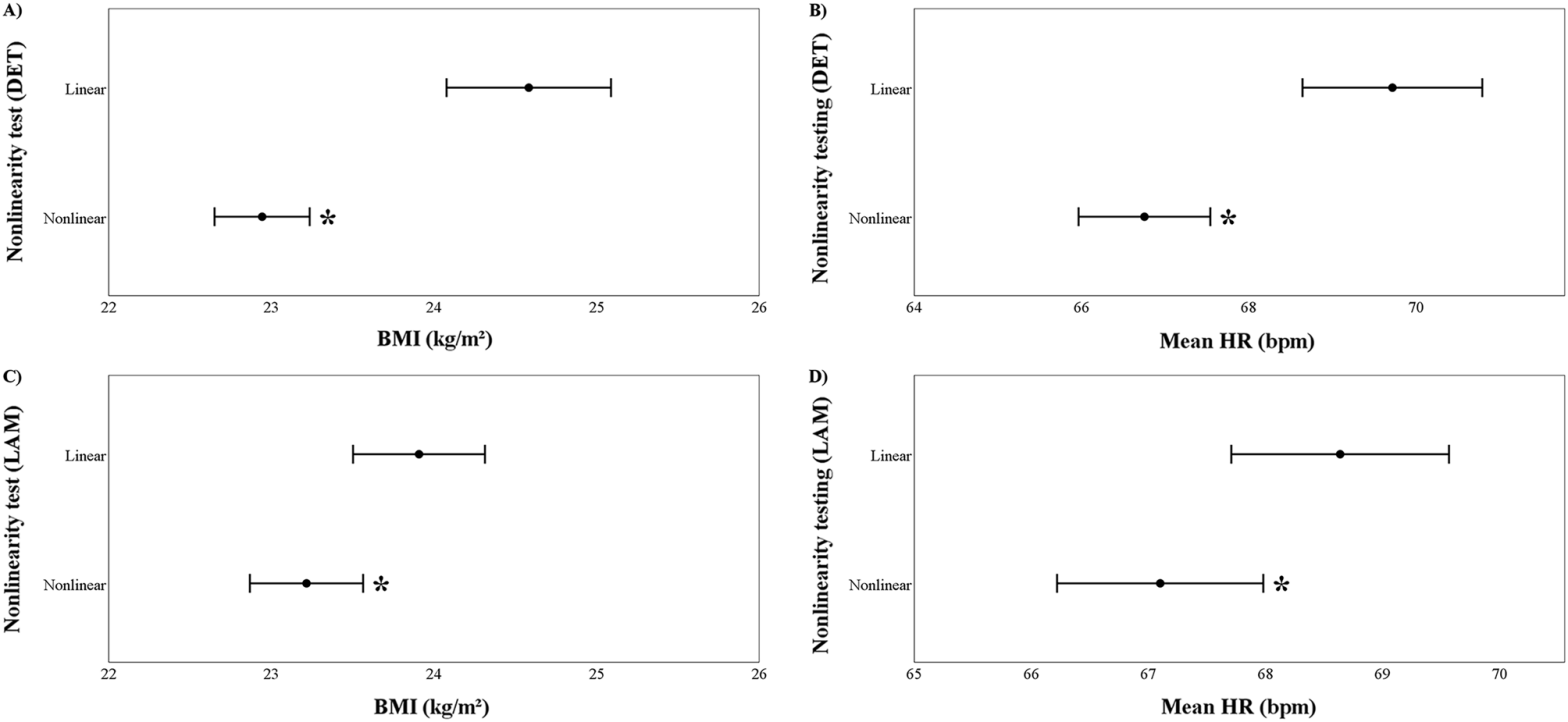
BMI and HR in linear and nonlinear time series. *p < 0.05.

Figure 6 shows that subjects that display a nonlinear behavior in accordance with the HRV nonlinear measures DET and LAM have both significantly smaller BMI and lower HR in comparison with those classified with linear HRV time series.

**Figure 7:**
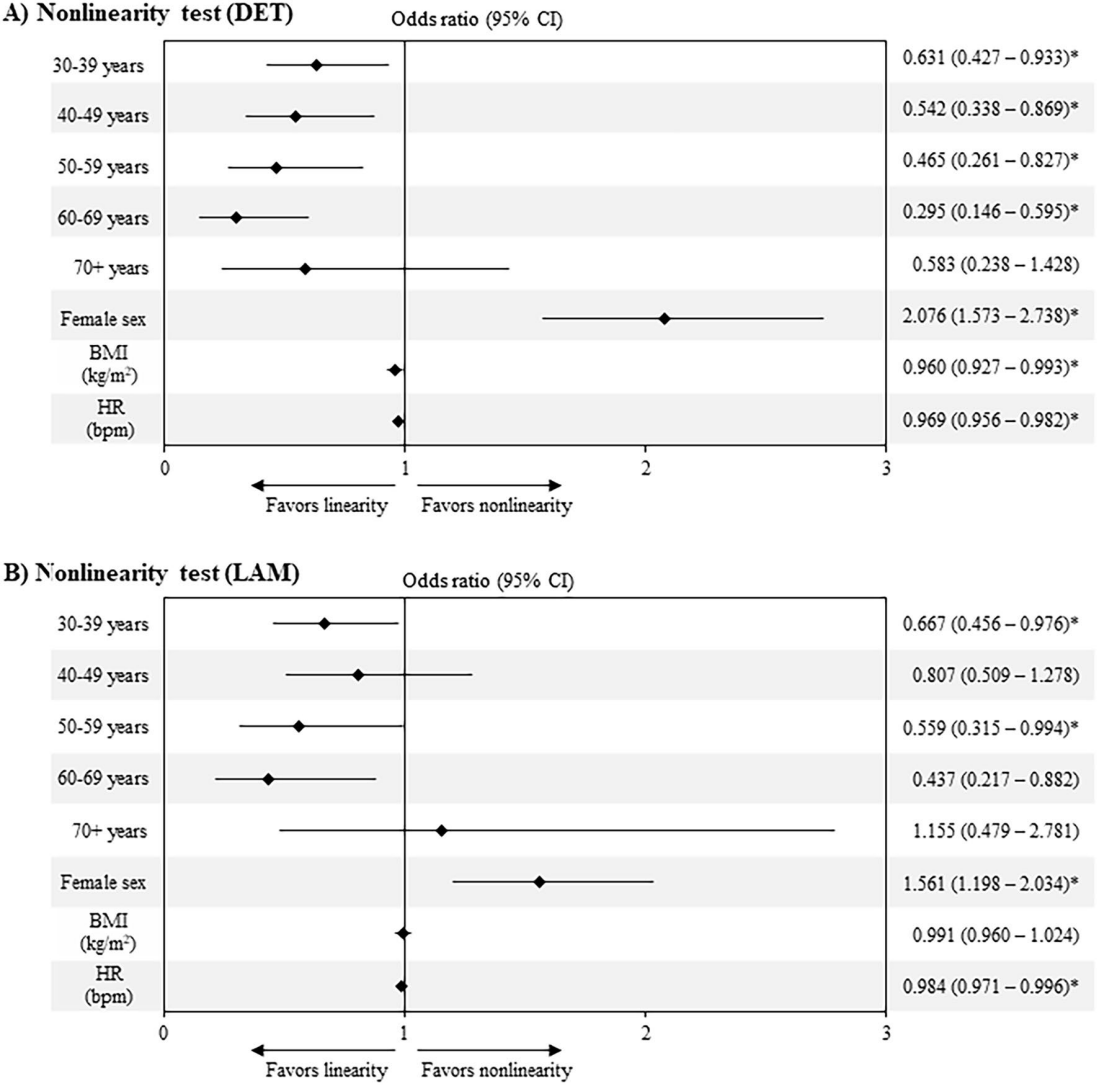
Effect of age group, female sex, BMI and HR in nonlinearity detection. *p < 0.05.

When DET is used as the discriminating statistic, the probability of finding nonlinearity in the HRV time series decreases with age, higher BMI, and HR (OR per unit shown in Fig. 7). Female sex increases the probability of classifying HRV time series as nonlinear. Similar results are found with LAM. However, for the 40–49 years of age group, BMI and HR do not show a statistically significant association to the presence of nonlinearity according to the OR.

## Discussion

In this work, we show that nonlinear information in HRV data is less likely to be found in older healthy humans as well as in subjects with larger BMI and higher HR. Also, female subjects are more likely to present nonlinear behavior in HRV. In a previous work we have shown that it is possible to dissect nonlinear components in physiological time series obtained from ECG recordings in healthy subjects and end-stage renal disease patients^14^ by employing a surrogate data method based on wavelets. Although several studies have applied surrogate data testing to investigate the presence of nonlinear dynamics in HRV, the effect of basic physical characteristics of human subjects such as sex, age, BMI and HR have not been fully assessed. This work provides a common background for the contextualized interpretation of nonlinear testing and shows that nonlinear information in HRV decreases through clinically healthy aging.

The exploration of nonlinear dynamics in HRV has been performed using methods that either take into consideration or not the influence of nonstationarity^14–18^. Previously we confirmed that linear nonstationary HRV time series may be erroneously reported as nonlinear in case of not using algorithms, such the GWR selected here, to generate surrogates that preserve the statistical properties, power spectral density distribution and nonstationarity of the original time series^14^.

Nonlinear behavior in HRV is attributed to the continuous interaction among the activities of the two branches of the autonomous nervous system: sympathetic and parasympathetic; the neuroendocrine system; the intrinsic cardiac nervous system; and the central pathways controlling the spontaneous beat-to-beat dynamics of heart rate. These interactions are expected to be nonlinear because of the different timing and hierarchical organization in addition to various physiological conditions involved in regulation that are based on the dynamic and simultaneously changing activity of the physical environmental stressors^29^.

Previous studies have reported that some complexity measures decay during healthy aging process^2,30^, which are proposed to reflect a decline of physiological dynamics^31,32^. However, it has been pointed out by other researches that decreasing values of nonlinear measures in physiological time series do not necessarily imply a loss of “complexity” in the data, and it is, in fact, much more complicated to link this phenomenon with physiological restrictions^9^, because the physiological behavior is not necessarily fully imprinted in corresponding time series^33^, and such measures may be biased by the context and characteristics of the recordings^9^. Further research on fractal properties of cardiac dynamics showed that despite identifying that the variability of the statistical dispersion of the heartbeats duration measured by the standard deviation is more restricted for older humans, the temporal fractal and nonlinear organization of HRV remains manifested in older but healthy individuals^8^.

The above-mentioned investigations are broadly heterogeneous in their methodology and studied subjects. In the present work, we present the analysis of a large sample size and wide age range, and also observe a progressive reduction in the statistical dispersion of HRV data and nonlinear measures values (DET and LAM), as observed by other authors^2,30^. However, this constriction on the age range does not mean a “complexity reduction” of the data^8^; therefore we applied a robust surrogate data algorithm that is a suitable tool for the presence of nonlinearity in each of the HRV time series^14^. This surrogate data testing shows that the proportion of time series that are classified as nonlinear under the hypothesis constructed by GWR is reduced with advanced age, and also by the biological sex of individuals. Moreover, we show that BMI, as a proxy indicator of body fatness, is also indirectly associated with nonlinearity contents. This may suggest that the metabolic status of individuals plays a role in the dynamical organization of HRV. Interestingly, the mean HR (i.e. the dynamic setting point of the HRV time series) has also a major impact on the intricate organization of the time series. It is known that the metabolic (long-term) and autonomic (short-term) regulations are strong modulators of HRV^34,35^. Nevertheless, the observation here of a connection between cardiometabolic indicators and nonlinear organization is quite relevant.

Our results are yet far from a full characterization of the HRV dynamical behavior in healthy aging and therefore of the cardiovascular system by itself. Nonlinearity testing may give different results because it is aimed to identify other nonlinear features^13^. Although age, sex, BMI and HR are independent factors that impact nonlinear testing, the biological mechanisms by which this relation is manifested can only be speculated and require further research in controlled experimental settings. Whether the length of the ECG recordings and daytime in which they were obtained also affect this relation remains unknown, and it is feasible that a given nonlinear feature requires larger length of data to be appreciated, or it could be intermittent thereby needing an appropriate moment to be observed. A future perspective of the current study is the investigation of the presence of nonlinear information in HRV data under pathologic conditions; this approach would allow obtaining a more detailed insight of the involved physiological mechanism related to the HRV time series nonlinear organization.

The limited data about subjects limits the generalization of the presented results to the vast myriad of physiological possibilities that may affect HRV. The database used lacks information about menopausal status or hormonal contraceptive use, which is a factor that has an important effect on HRV of adult women^36^. Also, the proportion of subjects > 70 years old is markedly small compared with the remaining groups, this is a potential selection bias and may underrepresent the HRV behavior for this age group.

## Conclusion

Nonlinear information as assessed by recurrence plot quantification analysis in HRV data is less likely to be found in older healthy humans as well as in subjects with larger BMI and higher HR. Also, female subjects are more likely to present HRV nonlinear behavior regardless of the age group.

### Supplementary Information

Supplementary Information.

## Data Availability

The data used in this study is publicly available at: https://physionet.org/content/autonomic-aging-cardiovascular/1.0.0/.

## Code availability

The used in this study is publicly available. Surrogate data testing: https://sites.google.com/site/chriskeylocknet/software/surrogate-generation-algorithms/pwiaaft. Recurrence plot analysis: https://tocsy.pik-potsdam.de/CRPtoolbox/.
