# Supplementary material for: Revisiting nonlinearity of heart rate variability in healthy aging
Source: Sci Rep. 2023 Aug 14;13:13185. doi: 10.1038/s41598-023-40385-1 (PMC10425345; doi:10.1038/s41598-023-40385-1)
Supplement: Supplementary file 1 — Supplementary Information. [file 41598_2023_40385_MOESM1_ESM.docx]

**Supplementary Information**

**Revisiting nonlinearity of heart rate variability in healthy aging**

Martín Calderón-Juárez^1, 2^, Gertrudis Hortensia González-Gómez^3^, Juan C. Echeverría^4^, and Claudia Lerma^2,*^

*Iterative amplitude adjusted Fourier transform (IAAFT)*

1. Compute the DFT in the original set of data $x_{n}$ and perform a random shuffle of data $x_{n}^{(0)}$.
2. Compute DFT from the shuffled data $x_{n}^{(0)}$ and replace the squared amplitudes with those obtained from original data $x_{n}$, and invert the transformation with the replaced amplitudes. This step permits to preserve the power spectrum with random phases.
3. Replace the values in the new set of data $x_{n}^{(i)}$ by the ones in the original data $x_{n}$ using a rank-order matching process. This step allows the data to have the same values as the original.
4. Repeat steps 2 and 3 until convergence; the reordering of data does not suffer further modifications, or these are too small. At the end of this algorithm, the power spectrum and distribution of data are very similar to the original.

*Gradual wavelet reconstruction (GWR)*

1. Use MODWT to decompose the original time series in a set of wavelet coefficients and determine the value (ρ) for the pinned coefficients.
2. For unpinned wavelet coefficients, apply IAAFT. For pinned wavelet coefficients:
3. Use the cubic Hermitian polynomial method to fit an exact interpolator through pinned coefficients and end values.
4. Random shuffle the unpinned coefficients and add the previous function and apply the IAAFT algorithm until convergence.
5. Perform inverse MODWT.
6. Repeat steps 2 and 3 of the IAAFT algorithm to match the power spectrum and data distribution.
